# Supplementary material for: Form and function of actin impacts actin health and aging
Source: bioRxiv. 2025 Nov 23:2025.11.22.689949. Preprint. [Version 1] doi: 10.1101/2025.11.22.689949 (PMC12667984; doi:10.1101/2025.11.22.689949)
Supplement: 1 [file NIHPP2025.11.22.689949V1-supplement-1.pdf]

bioRxiv preprint doi: https://doi.org/10.1101/2025.11.22.689949; this version posted November 23, 2025. The copyright holder for this preprint (which was not certified by peer review) is the author/funder, who has granted bioRxiv a license to display the preprint in perpetuity. It is made available under aCC-BY 4.0 International license.

Supplemental Figures

bioRxiv preprint doi: https://doi.org/10.1101/2025.11.22.689949; this version posted November 23, 2025. The copyright holder for this preprint (which was not certified by peer review) is the author/funder, who has granted bioRxiv a license to display the preprint in perpetuity. It is made available under aCC-BY 4.0 International license.

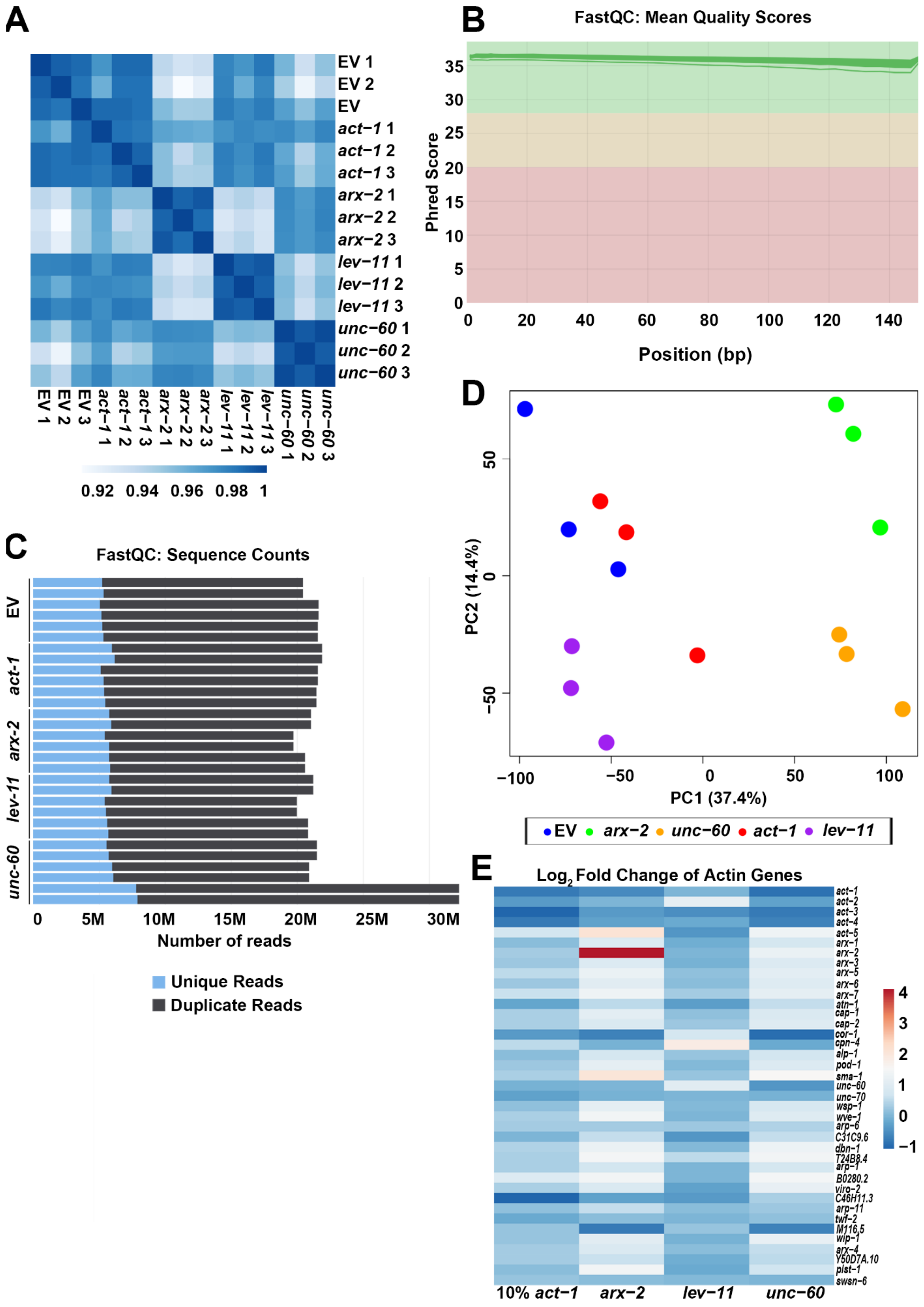

bioRxiv preprint doi: https://doi.org/10.1101/2025.11.22.689949; this version posted November 23, 2025. The copyright holder for this preprint (which was not certified by peer review) is the author/funder, who has granted bioRxiv a license to display the preprint in perpetuity. It is made available under aCC-BY 4.0 International license.

Fig. S1: Quality control for RNA-seq libraries of C. elegans exposed to actin and ABP knockdown. (A) Spearman correlation plot of all RNA sequencing libraries. **(B)** Mean quality score (Phred score) of each RNA sequencing library. X-axis and Y-axis indicate the base pair position of each sequence and the Phred score, respectively. The graph was generated by MultiQC tool[189]. **(C)** The number of unique (blue) and duplicated (grey) reads from each pair-wise sequencing library (n=3). **(D)** PCA plots of actin and ABP knockdown in *C. elegans*. **(E)** Heatmap of log2(fold changes) for all genes annotated as cytoskeleton: Actin function in WormCat[190]. See **Table S9** for expression details of the genes used in the heatmap.

bioRxiv preprint doi: https://doi.org/10.1101/2025.11.22.689949; this version posted November 23, 2025. The copyright holder for this preprint (which was not certified by peer review) is the author/funder, who has granted bioRxiv a license to display the preprint in perpetuity. It is made available under aCC-BY 4.0 International license.

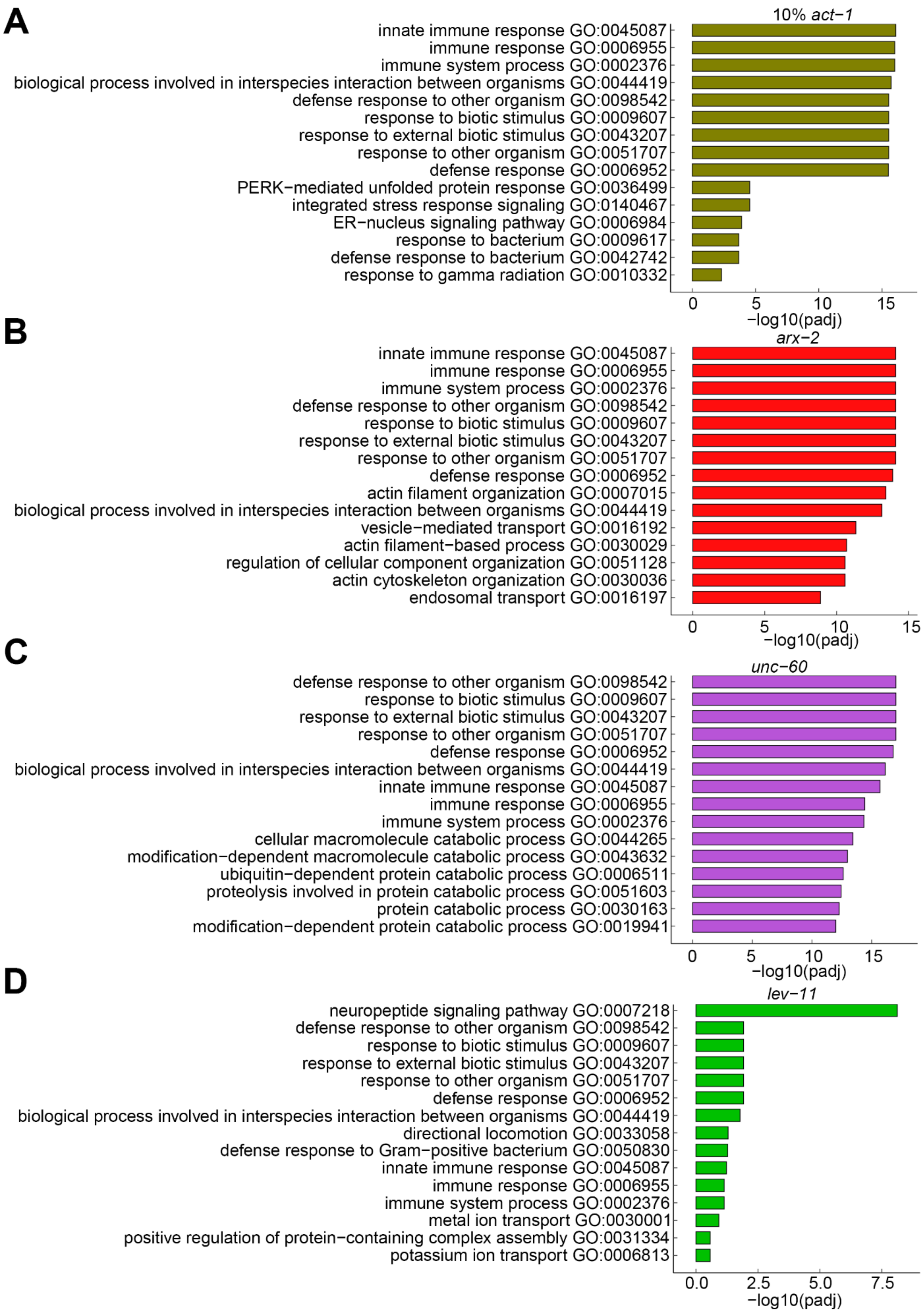

bioRxiv preprint doi: https://doi.org/10.1101/2025.11.22.689949; this version posted November 23, 2025. The copyright holder for this preprint (which was not certified by peer review) is the author/funder, who has granted bioRxiv a license to display the preprint in perpetuity. It is made available under aCC-BY 4.0 International license.

Fig. S2. Gene ontology analysis of actin disruption.
GO analysis of unique DEGs not shared with another condition for **(A)** *act-1,* **(B)** *arx-2***, (C**) *unc-60* and **(D)** *lev-11*. All DEGs were selected for adj p-val < 0.05, and the GOs were biological processes (BP) with q-value < 0.5. See **Table S5** for Go IDs, genes and statistics of the GO Analysis.

bioRxiv preprint doi: https://doi.org/10.1101/2025.11.22.689949; this version posted November 23, 2025. The copyright holder for this preprint (which was not certified by peer review) is the author/funder, who has granted bioRxiv a license to display the preprint in perpetuity. It is made available under aCC-BY 4.0 International license.

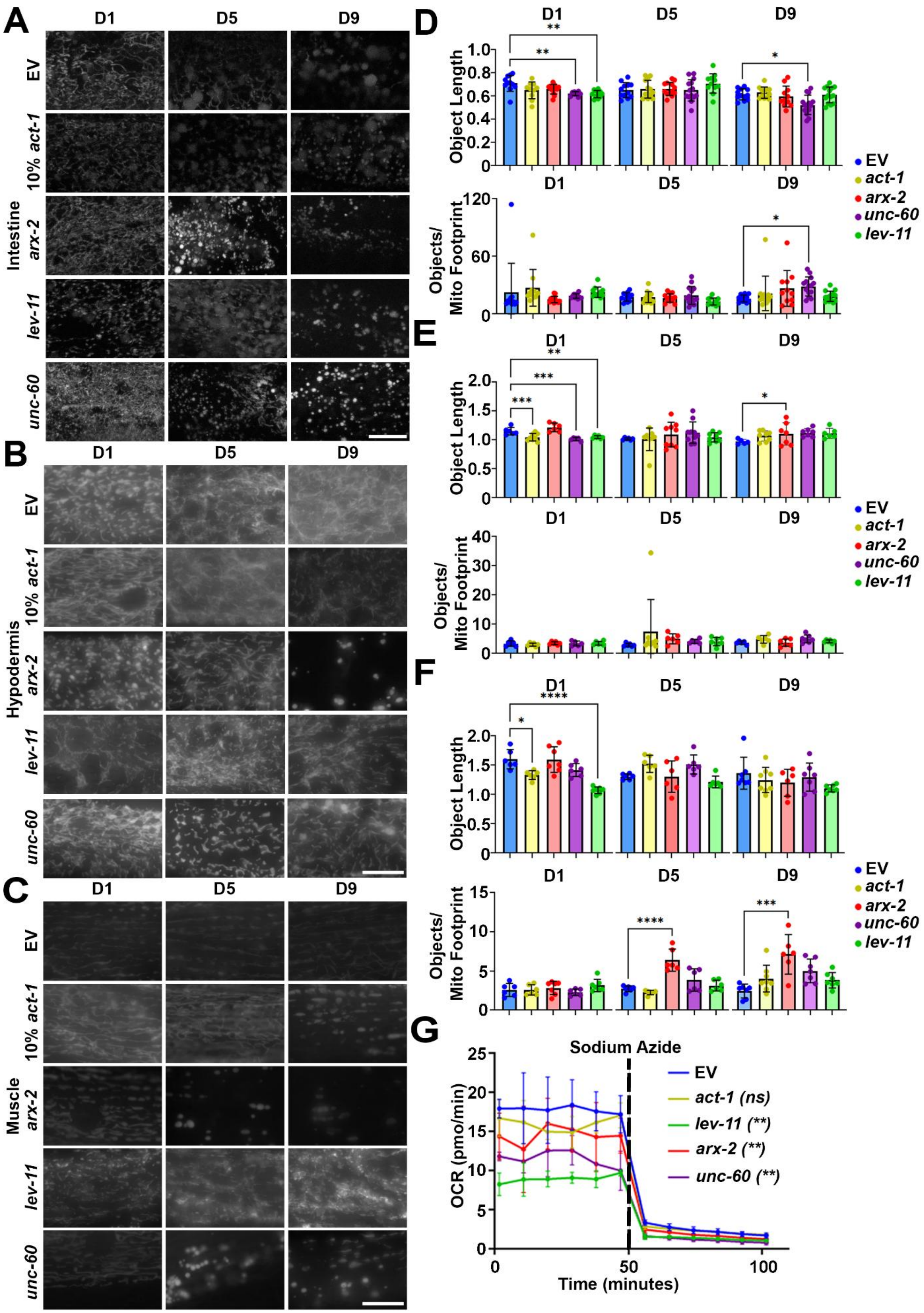

bioRxiv preprint doi: https://doi.org/10.1101/2025.11.22.689949; this version posted November 23, 2025. The copyright holder for this preprint (which was not certified by peer review) is the author/funder, who has granted bioRxiv a license to display the preprint in perpetuity. It is made available under aCC-BY 4.0 International license.

Fig. S3: Actin disruption leads to mitochondrial dysfunction. Animals expressing *vha-6p::MLS::GFP* **(A)**, *col-19p::MLS::GFP* **(B)**, and *myo-3p::MLS::GFP* **(C)**, were grown on empty vector (EV), a 1:9 mix of *act-1*/EV (10% *act-1*), *arx-2*, *lev-11*, or *unc-60* RNAi from hatch and imaged during days 1, 5, and 9 of adulthood. **(D, E, F)** All quantification was performed using mitoMAPR. Object length and objects/mitochondrial footprint are shown here as example measurements, and all mitochondrial measurements measured by mitoMAPR are available in **Table S8**. In mitoMAPR-based quantification, the "objects/mitochondrial footprint" refers to the total number of objects detected as mitochondria and the area occupied by all the mitochondria within a defined region of interest**. (G)** Seahorse analysis of mitochondrial respiration/OCR (pmol/min) of day 1 wild type N2 animals grown on empty vector (EV, blue), 1:9 ratio of *act-1*:EV RNAi (yellow), *arx-2* (red), *lev-11* (green), or *unc-60* (purple) RNAi from hatch. 50 mM sodium azide was applied to measure non-mitochondrial respiration. Scale bar is 10 μm.

bioRxiv preprint doi: https://doi.org/10.1101/2025.11.22.689949; this version posted November 23, 2025. The copyright holder for this preprint (which was not certified by peer review) is the author/funder, who has granted bioRxiv a license to display the preprint in perpetuity. It is made available under aCC-BY 4.0 International license.

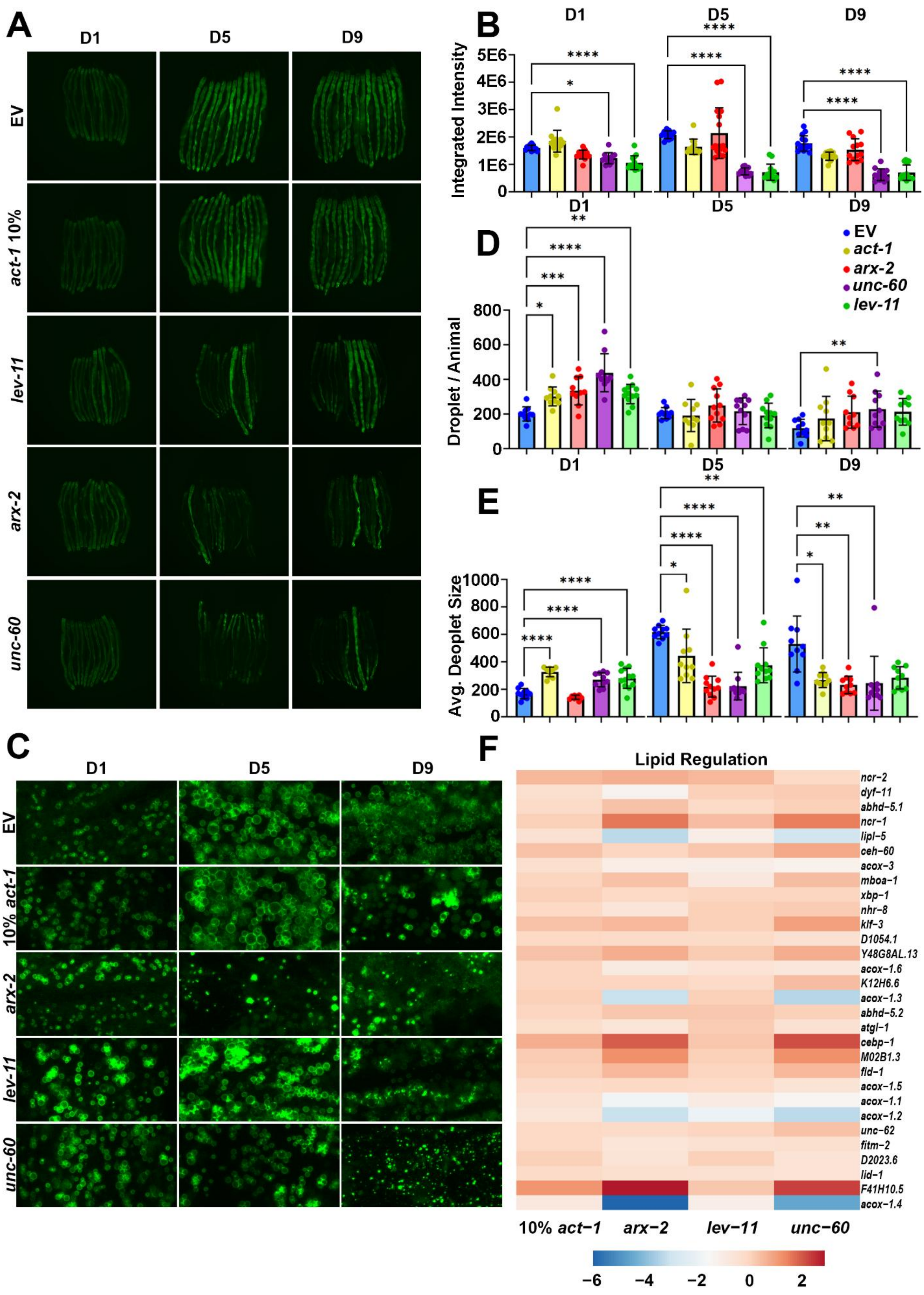

bioRxiv preprint doi: https://doi.org/10.1101/2025.11.22.689949; this version posted November 23, 2025. The copyright holder for this preprint (which was not certified by peer review) is the author/funder, who has granted bioRxiv a license to display the preprint in perpetuity. It is made available under aCC-BY 4.0 International license.

Fig. S4: Actin disruption reduces lipid droplet size during aging. (A) Representative fluorescent stereomicroscope images of lipid droplets by visualization of DHS-3::GFP. Animals were grown on empty vector (EV), a 1:9 mix of *act-1*/EV (10% *act-1*), *arx-2*, *lev-11*, or *unc-60* RNAi from hatch. All animals were imaged on day 1, 5, and 9 of adulthood. **(B)** Quantification of GFP signal as measured by integrated intensity, based on 3 biological replicates of ≥ 12 animals per experimental condition. Two-way ANOVA was used for each time point. **(C)** Representative fluorescent confocal images of lipid droplets by visualization of DHS-3::GFP. Animals were grown on empty vector (EV), a 1:9 mix of *act-1*/EV (10% *act-1*), *arx-2*, *lev-11*, or *unc-60* RNAi from hatch. All animals were imaged on day 1, 5, and 9 of adulthood. **(D)** Quantification of the number of lipids per animal, based on 3 biological replicates of ≥ 4 animals per experimental condition. Two-way ANOVA was used for each time point. **(E)** Quantification of the average lipid size (in pixels) in each animal, based on 3 biological replicates of ≥ 4 animals per experimental condition. Two-way ANOVA was used for each time point. **(F)** Heat map of gene expression changes involved in lipid homeostasis (GO:0055088). See **Table S9** for expression details of the genes used in the heatmap.

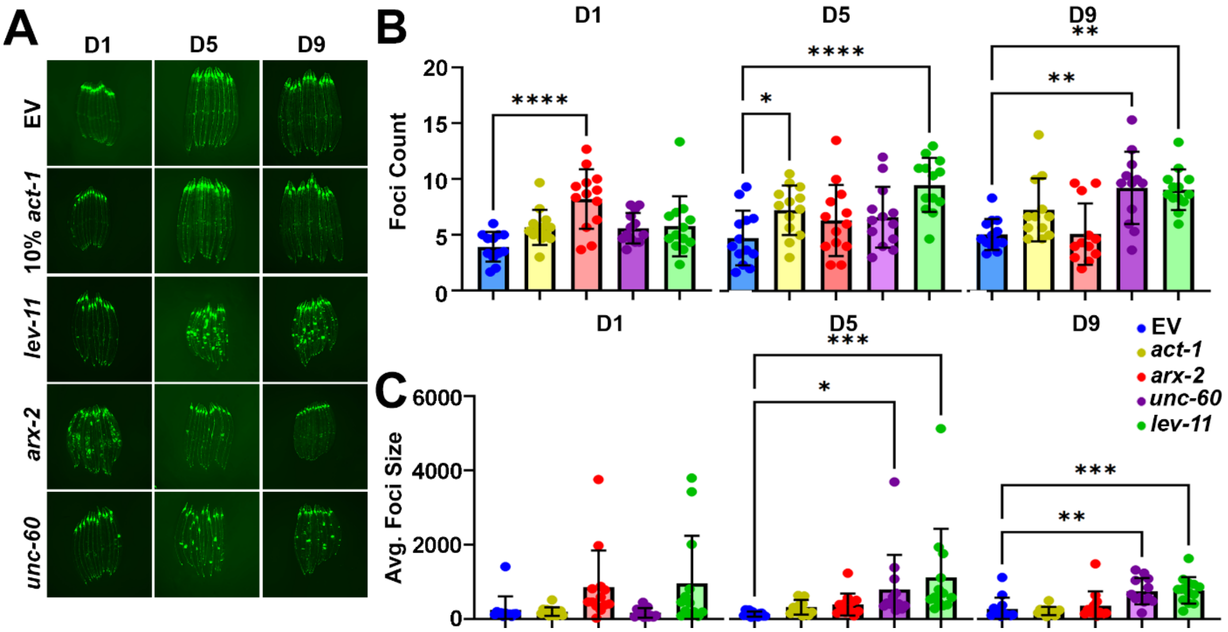

Fig. S5. Actin disruption leads to dysregulation of proteostasis.
(A) Representative stereomicroscope images of protein aggregation in animals expressing neuronal polyglutamine repeats (*rgef-1p::polyQ40::YFP*) at Day 1, Day 5 and Day 9 of adulthood. Animals were grown on empty vector (EV), a 1:9 mix of *act-1*/EV (10% *act-1*), *arx-2*, *lev-11*, or *unc-60* RNAi from hatch. Quantification of the **(B)** total number of aggregates and **(C)** average aggregate size was based on 3 biological replicates of ≥ 12 animals per experimental condition. Two-way ANOVA was used for each time point.

bioRxiv preprint doi: https://doi.org/10.1101/2025.11.22.689949; this version posted November 23, 2025. The copyright holder for this preprint (which was not certified by peer review) is the author/funder, who has granted bioRxiv a license to display the preprint in perpetuity. It is made available under aCC-BY 4.0 International license.

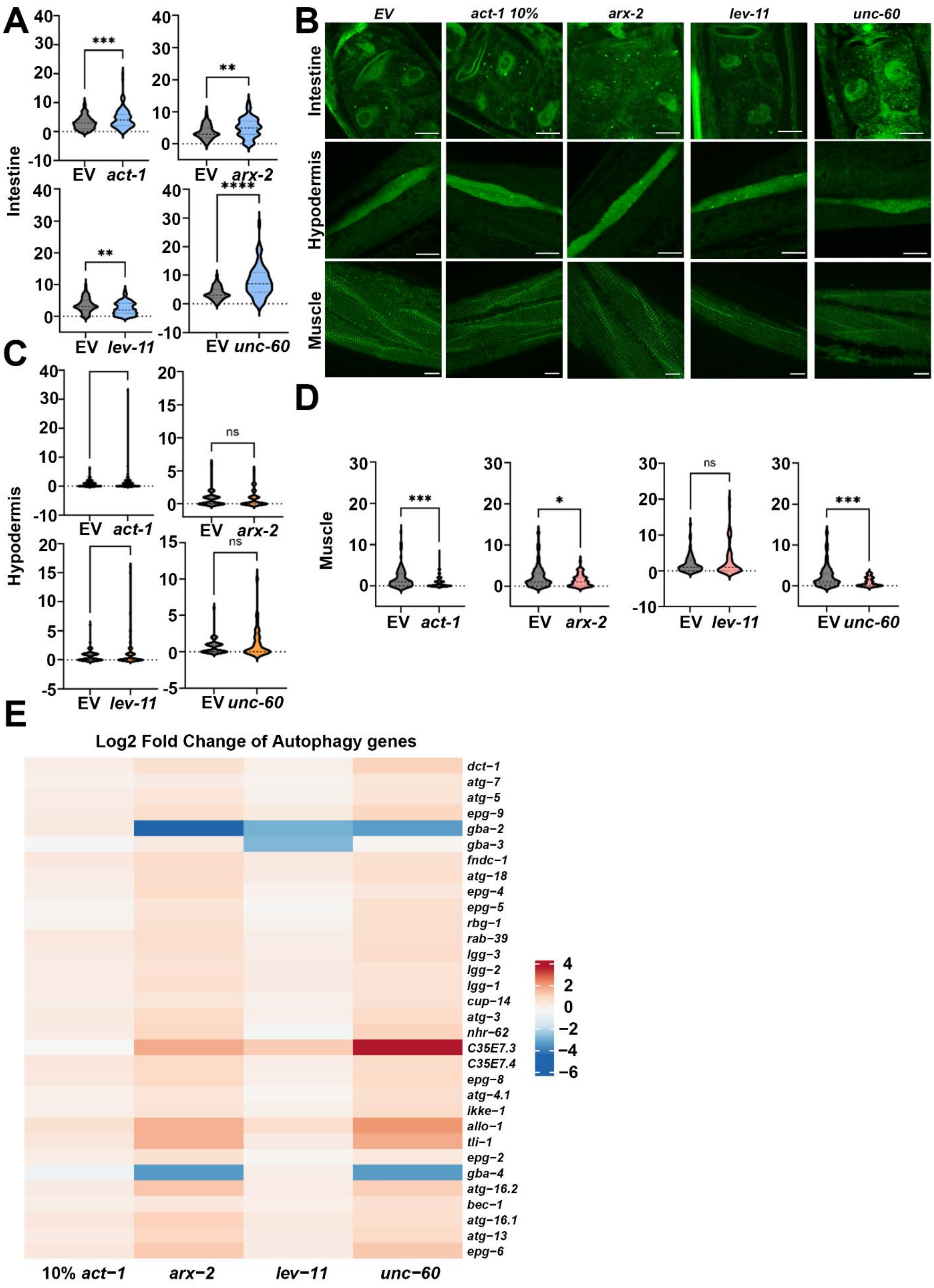

bioRxiv preprint doi: https://doi.org/10.1101/2025.11.22.689949; this version posted November 23, 2025. The copyright holder for this preprint (which was not certified by peer review) is the author/funder, who has granted bioRxiv a license to display the preprint in perpetuity. It is made available under aCC-BY 4.0 International license.

Fig. S6: Actin disruption leads to tissue-specific dysregulation of autophagy (B) Representative images of GFP::LGG-1 puncta in intestinal, hypodermal seam and muscle cells of control and RNAi-treated animals targeting *act-1, arx-2, unc-60, or lev-11* from hatch. Violin plots show quantification of GFP::LGG-1 puncta in **(A)** intestinal cells (CTRL, N = 63-107; *act-1*, N = 113; *arx-2*, N = 63; *unc-60*, N = 71; *lev-11*, N = 74 cells), **(C)** hypodermal seam cells (CTRL, N = 84–87; *act-1*, N = 87; *arx-2*, N = 51; *unc-60*, N = 45; *lev-11*, N = 70 cells) and **(D)** muscle cells (CTRL, N = 84–88; *act-1*, N = 88; *arx-2*, N = 51; *unc-60*, N = 37; *lev-11*, N = 57 cells. Data are pooled from three to five independent experiments. Statistical significance was determined by Welch's two-tailed t-test (Graphpad). ns: $P > 0.05$, *$P < 0.05$, **$P < 0.01$, ***$P < 0.001$, ****$P < 0.0001$. Scale bars: 10 µm. **(E)** Heat map of the changes in expression ($p<0.5$) of genes involved in autophagy (directly annotated as "autophagy" in AmiGO2[188]). See **Table S9** for expression details of the genes used in the heatmap.

bioRxiv preprint doi: https://doi.org/10.1101/2025.11.22.689949; this version posted November 23, 2025. The copyright holder for this preprint (which was not certified by peer review) is the author/funder, who has granted bioRxiv a license to display the preprint in perpetuity. It is made available under aCC-BY 4.0 International license.

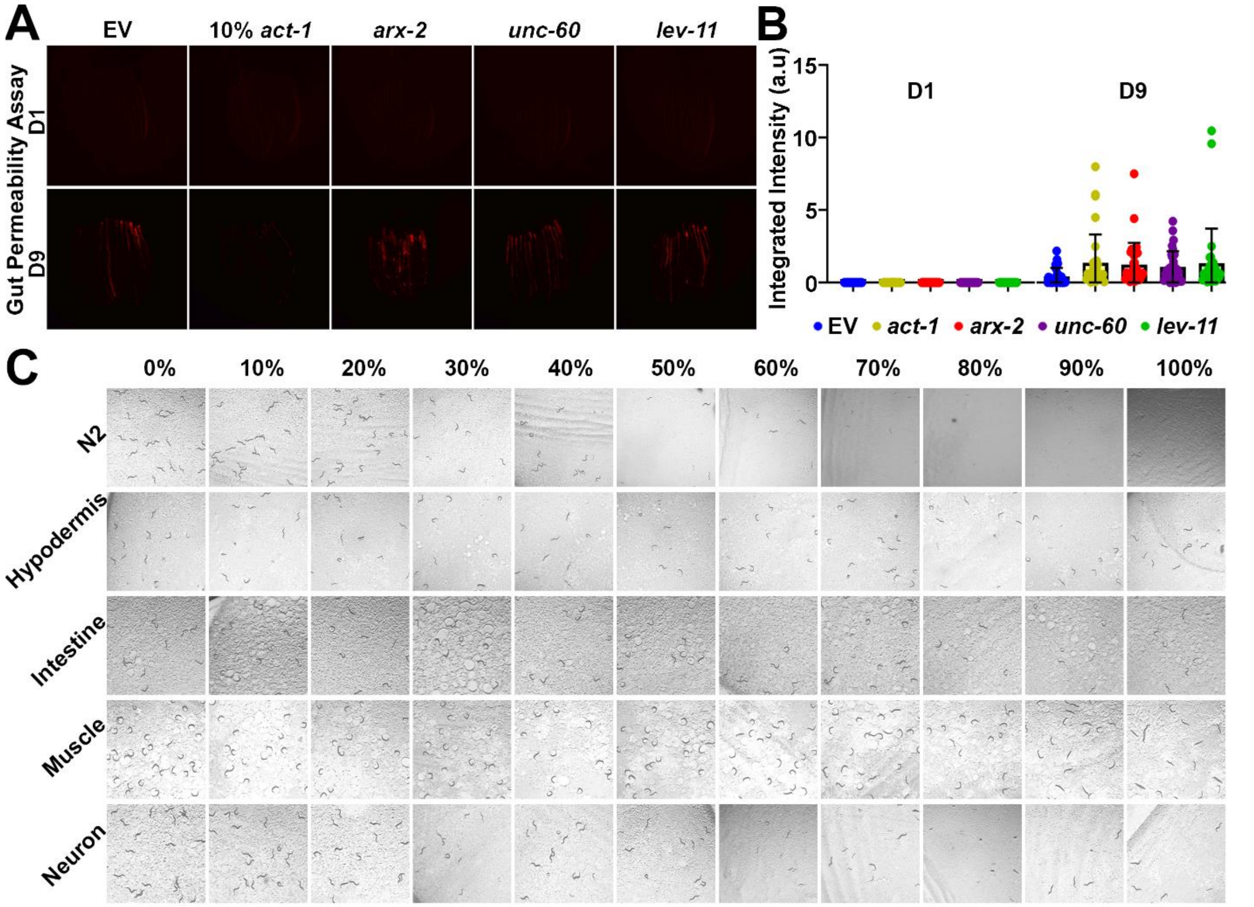

Fig. S7: Actin disruption leads to tissue-specific dysregulation of gut barrier integrity. **(A)** Representative stereoscope images of gut colonization at Day 1 and Day 9 in animals grown with EV, 10% *act-1*, *arx-2*, *lev-11*, or *unc-60* RNAi mixed with 20% HT115 bacteria driving mCherry expression. **(B)** Quantification of gut colonization fluorescence signal. 10-15 worms were imaged for two technical replicates for each of 3 independent biological replicates (with ≥ 150 worms total per replicate per strain/condition), analyzed via two-way ANOVA test. **(C)** Representative stereoscopic images of *C. elegans* exposed to varying dilutions 0%-100% of actin RNAi from L1 stage of development. X-axis indicates dilution percentage with 0% being no actin RNAi and 100% meaning non-diluted RNAi. Y-axis represents the tissue where RNAi is functional with N2 being the wild-type control with whole-body functioning RNAi machinery.

bioRxiv preprint doi: https://doi.org/10.1101/2025.11.22.689949; this version posted November 23, 2025. The copyright holder for this preprint (which was not certified by peer review) is the author/funder, who has granted bioRxiv a license to display the preprint in perpetuity. It is made available under aCC-BY 4.0 International license.

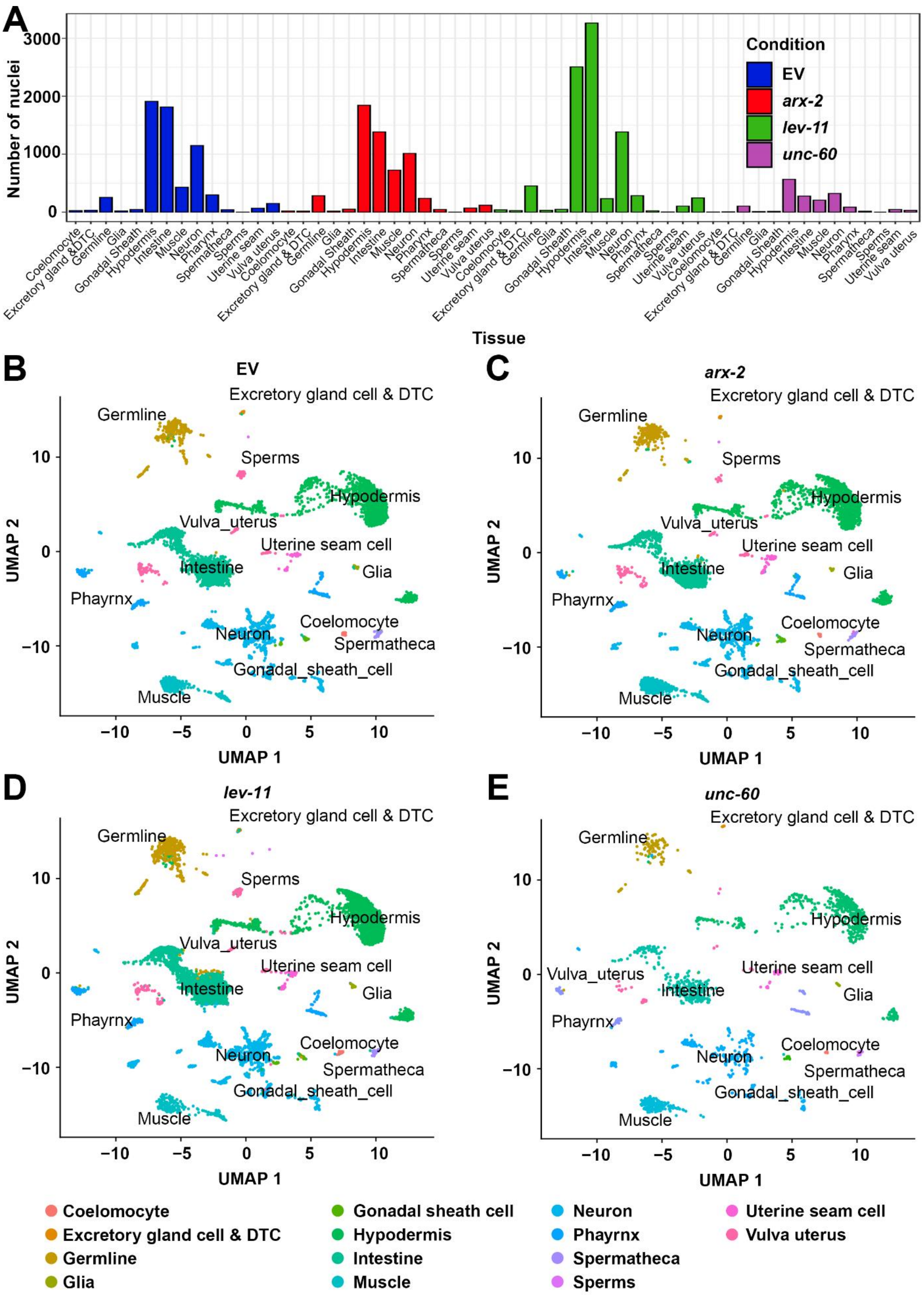

bioRxiv preprint doi: https://doi.org/10.1101/2025.11.22.689949; this version posted November 23, 2025. The copyright holder for this preprint (which was not certified by peer review) is the author/funder, who has granted bioRxiv a license to display the preprint in perpetuity. It is made available under aCC-BY 4.0 International license.

Fig. S8. Annotation and clustering of single-nuclei RNA sequencing. **(A)** Summary plot of all sequenced annotated nuclei for each tissue and condition. **(B-E)** UMAP of the data segmented to corresponding cell types, as identified by cell-type biomarkers in D1 **(B)** control animals and animals with **(C)** *arx-2*, **(D)** *lev-11*, and **(E)** *unc-60* knockdown.

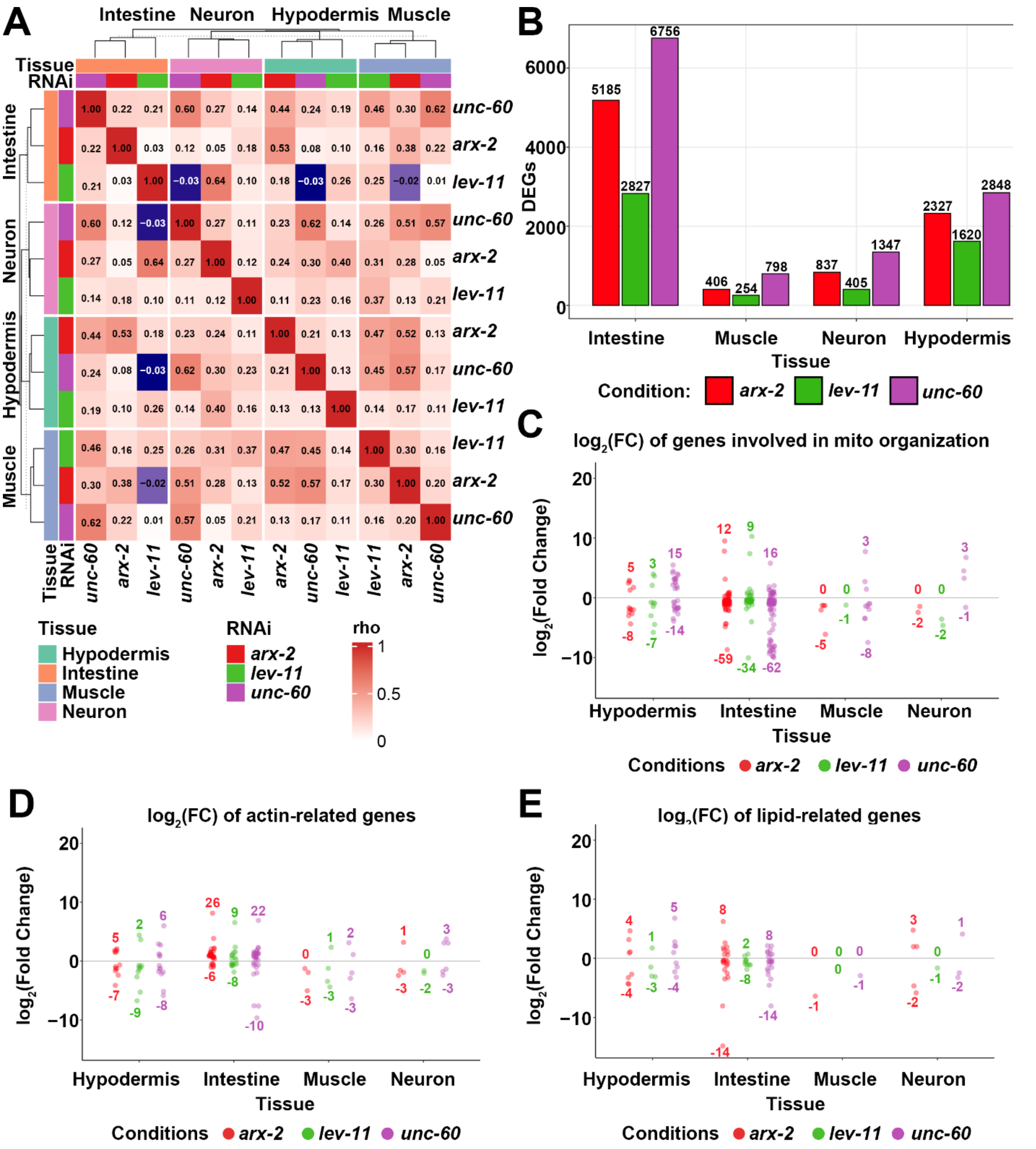

Fig. S9: Deconvolution supplemental information. (A) Clustering of all samples based on the Spearman correlation between every tissue and condition. Clustering is performed within each tissue slice, and dendrograms illustrate the similarity structure. See **Table S10** for Spearman correlations between each sample. **(B)** Bar plot showing the number of DEGs for each condition and key selected tissues: Intestine, Muscle, Neuron and Hypodermis. (**C-E**) Dot plots showing

bioRxiv preprint doi: https://doi.org/10.1101/2025.11.22.689949; this version posted November 23, 2025. The copyright holder for this preprint (which was not certified by peer review) is the author/funder, who has granted bioRxiv a license to display the preprint in perpetuity. It is made available under aCC-BY 4.0 International license.

significant DEGs involved in **(C)** mitochondrial organization (labeled as "involved in mitochondrion organization" in AmiGO2[188]), **(D)** actin maintenance (annotated as cytoskeleton: Actin function in WormCat) and **(E)** lipid regulation (GO:0055088) under ABP knockdown in 4 key tissues: Hypodermis, Intestine, Muscle and Neuron. See **Table S6** for DEGs in each dotplot.

bioRxiv preprint doi: https://doi.org/10.1101/2025.11.22.689949; this version posted November 23, 2025. The copyright holder for this preprint (which was not certified by peer review) is the author/funder, who has granted bioRxiv a license to display the preprint in perpetuity. It is made available under aCC-BY 4.0 International license.

A

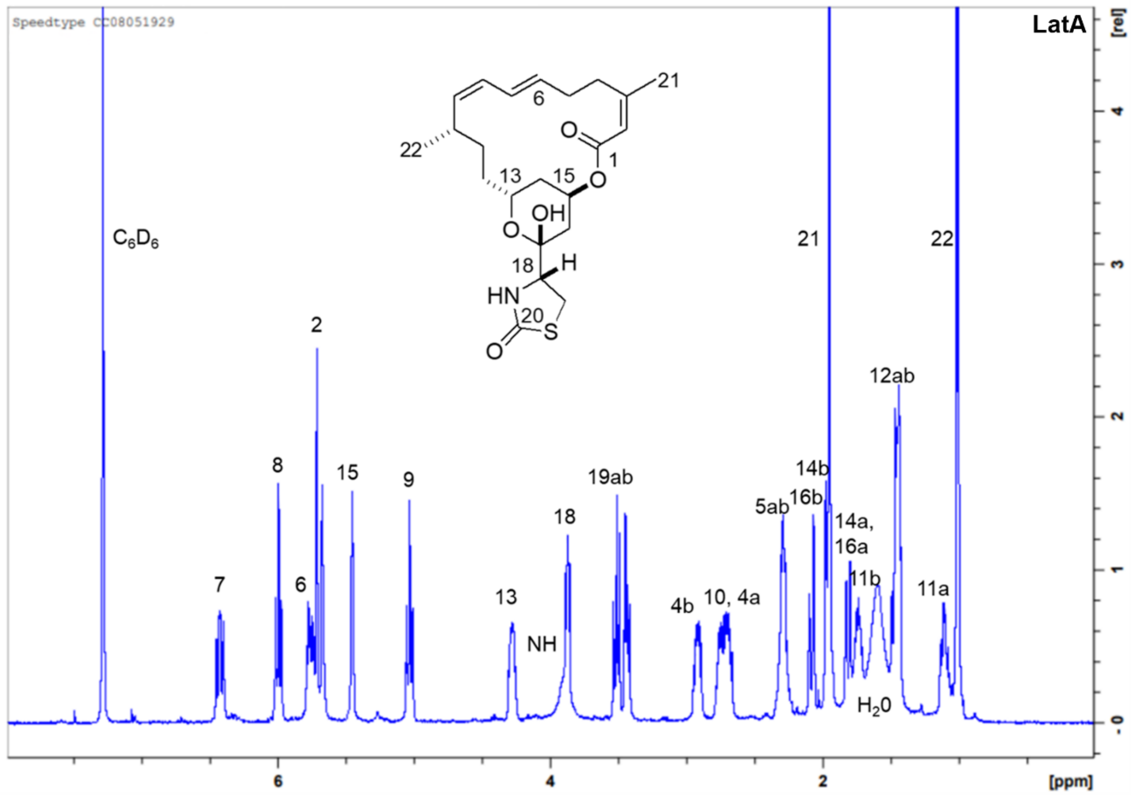

B

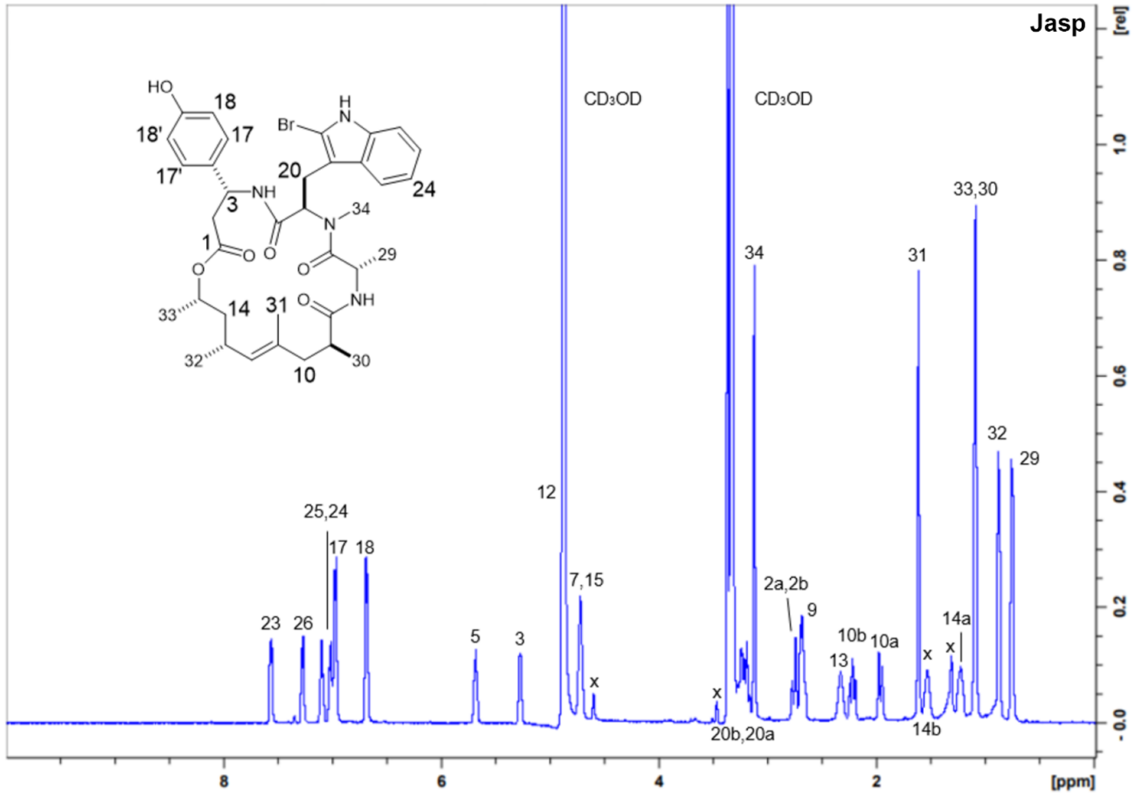

bioRxiv preprint doi: https://doi.org/10.1101/2025.11.22.689949; this version posted November 23, 2025. The copyright holder for this preprint (which was not certified by peer review) is the author/funder, who has granted bioRxiv a license to display the preprint in perpetuity. It is made available under aCC-BY 4.0 International license.

Fig. S10. NMR analysis of LatA and Jasp. ^{1}H NMR spectrum of **(A)** latrunculin A (LatA) in Benzene-*d6* at 500 MHz and **(B)** jasplakinolide (Jasp) in CD_3OD at 500MHz

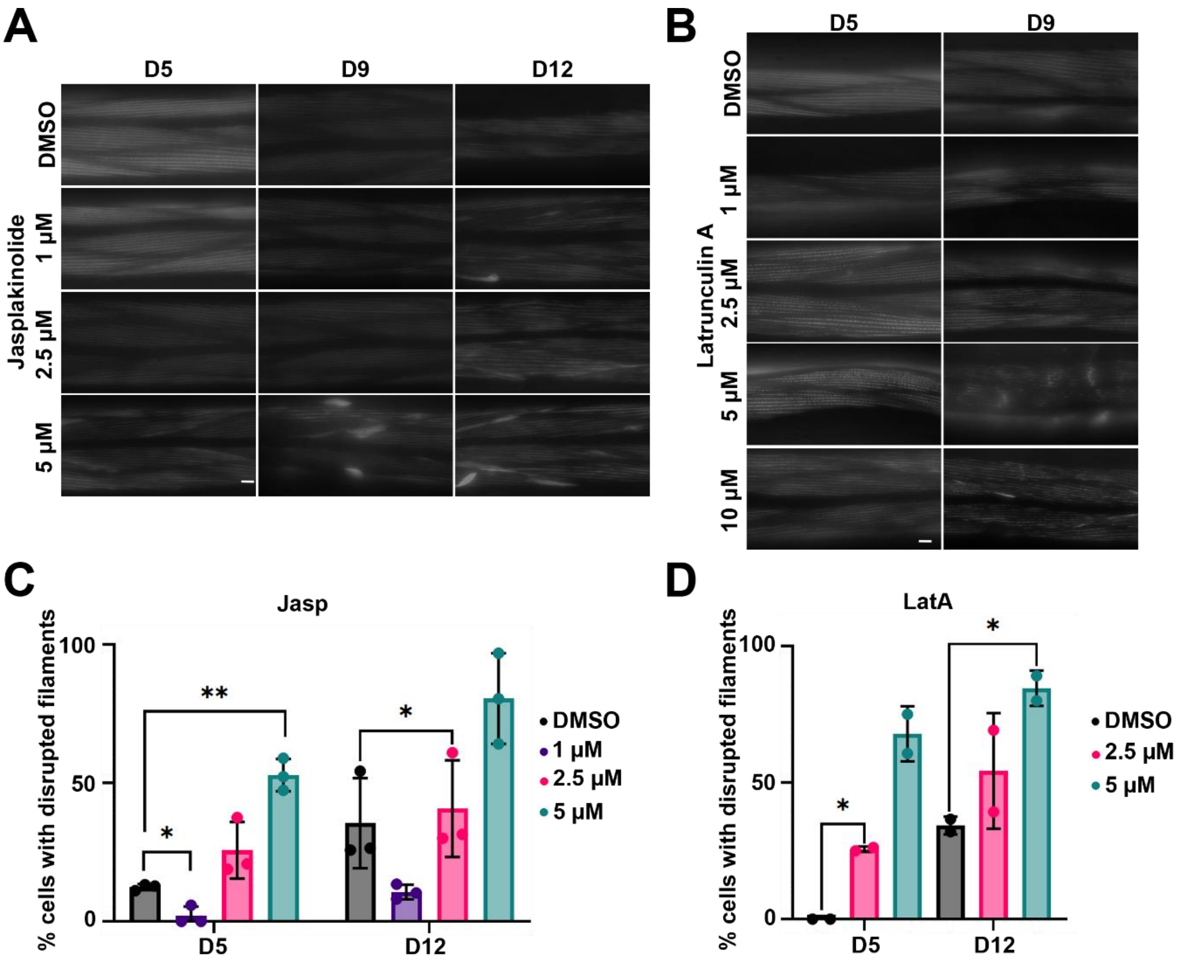

Fig. S11. Small-molecule actin disruption effect on healthspan. (A) Representative fluorescent images of adult animals expressing LifeAct::mRuby in the muscle grown on empty vector (EV) and varying concentrations of Jasplakinolide (1µM, 2.5 µM, 5 µM) or DMSO control. **(B)** Representative fluorescent images of adult animals expressing LifeAct::mRuby in the muscle grown on empty vector (EV) and varying concentrations of Latrunculin A (1µM, 2.5 µM, 5 µM, 10 µM) or DMSO control. All muscle images were captured on day 5, and 9 of adulthood and on a Leica Thunder Imager. **(C-D)** Quantification of actin structure quality in animals expressing LifeAct::mRuby in the muscle grown on empty vector (EV) and varying concentrations of **(C)** Jasplakinolide and **(D)** Latrunculin A (1µM, 2.5 µM, 5 µM) or DMSO control. N=3 n>5.

bioRxiv preprint doi: https://doi.org/10.1101/2025.11.22.689949; this version posted November 23, 2025. The copyright holder for this preprint (which was not certified by peer review) is the author/funder, who has granted bioRxiv a license to display the preprint in perpetuity. It is made available under aCC-BY 4.0 International license.

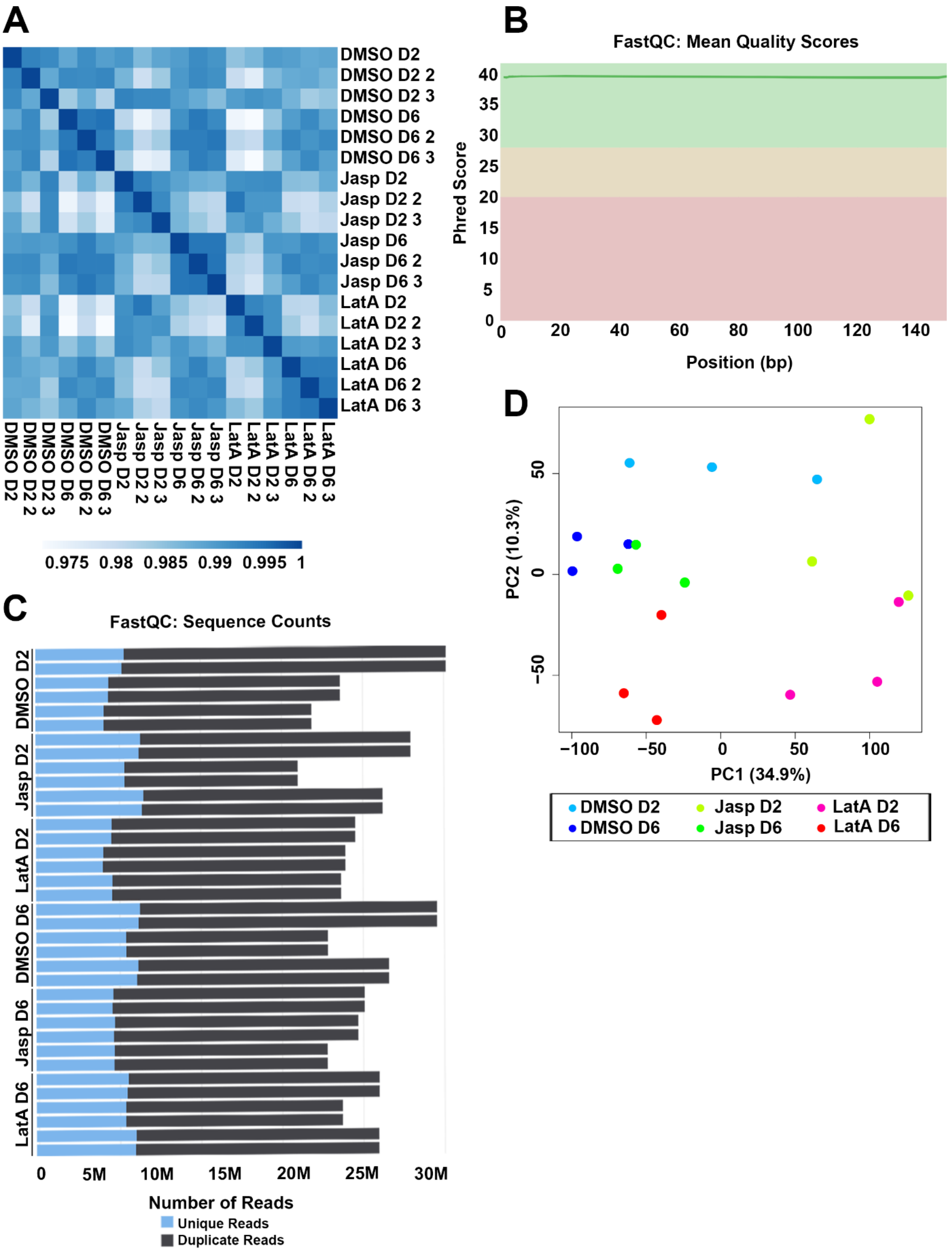

Fig. S12. Quality control of LatA and Jasp RNA-seq data. (A) Spearman correlation plot of all RNA sequencing libraries. **(B)** Mean quality score (Phred score) of each sequencing library.

bioRxiv preprint doi: https://doi.org/10.1101/2025.11.22.689949; this version posted November 23, 2025. The copyright holder for this preprint (which was not certified by peer review) is the author/funder, who has granted bioRxiv a license to display the preprint in perpetuity. It is made available under aCC-BY 4.0 International license.

X-axis and Y-axis indicate the base pair position of each sequence and the Phred score, respectively. The graph was generated by MultiQC tool[189]. **(C)** The number of unique (blue) and duplicated (grey) reads from each pair-wise sequencing library (n=3). **(D)** PCA plots of Jasplakinolide and Latrunculin A exposure in C. elegans.

bioRxiv preprint doi: https://doi.org/10.1101/2025.11.22.689949; this version posted November 23, 2025. The copyright holder for this preprint (which was not certified by peer review) is the author/funder, who has granted bioRxiv a license to display the preprint in perpetuity. It is made available under aCC-BY 4.0 International license.

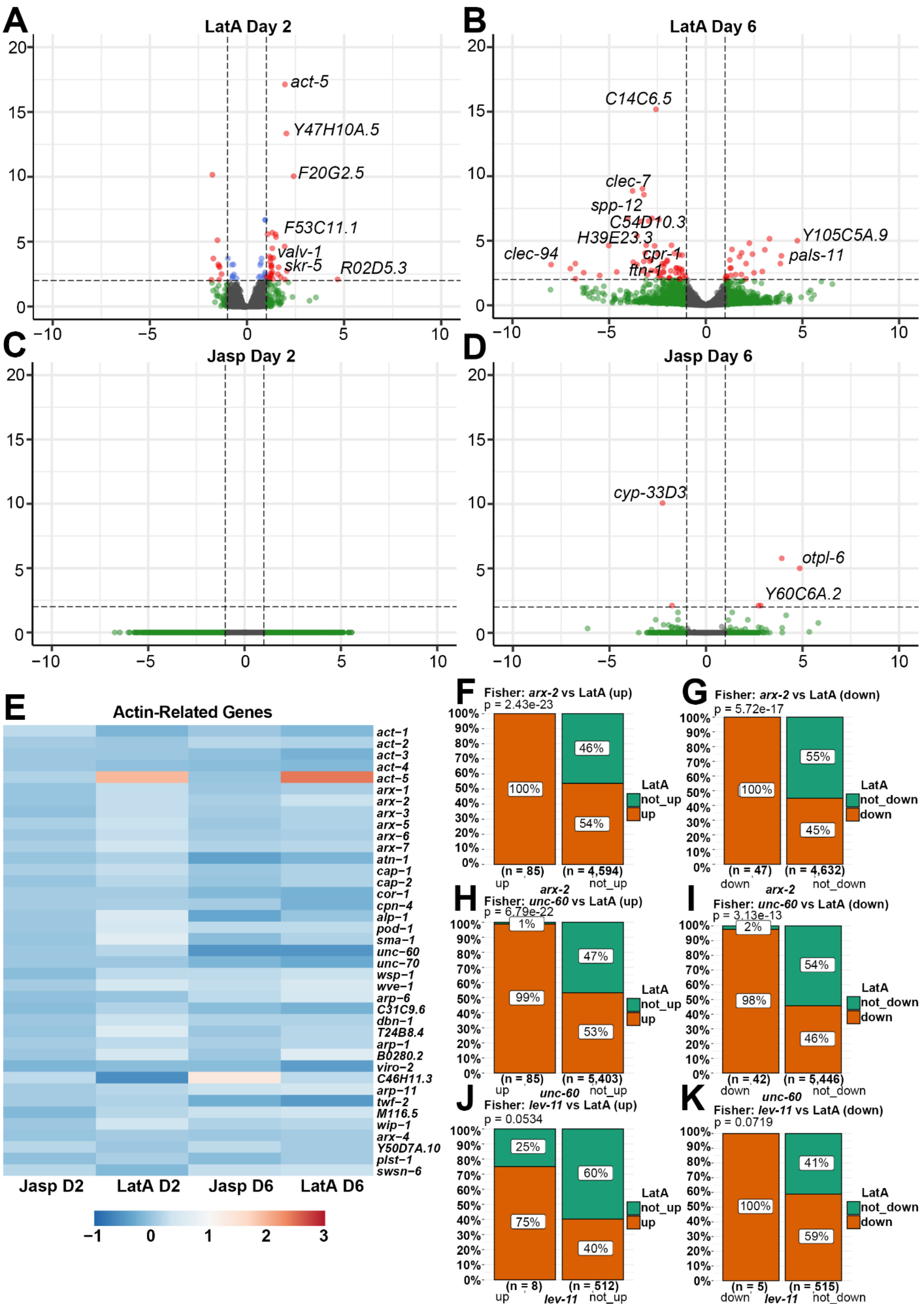

bioRxiv preprint doi: https://doi.org/10.1101/2025.11.22.689949; this version posted November 23, 2025. The copyright holder for this preprint (which was not certified by peer review) is the author/funder, who has granted bioRxiv a license to display the preprint in perpetuity. It is made available under aCC-BY 4.0 International license.

Fig. S13. Actin-binding small molecules induces a mild transcriptional response. Volcano plots of genome-wide changes in gene expression upon drug exposure from Day 1 of adulthood on LatA until **(A)** Day 2 and **(B)** Day 6, and Jasp until **(C)** Day 2 and **(D)** Day 6 of adulthood. Red dots indicate significantly differentially expressed genes with p-value ≤ 0.01 and $log_2FC>|2|$. Blue dots indicate significantly differentially expressed genes with p-value ≤ 0.01 and $log_2FC<|2|$. Green dots indicate significantly differentially expressed genes with p-value ≥0.01. See **Table S11** for a list of differentially expressed genes and expression values. **(E)** Heat map of differentially expressed genes annotated as “cytoskeleton: Actin function” in WormCat[190]. Warmer colors indicate increased expression, and cooler colors indicate decreased expression. See **Table S9** for expression details of the genes used in the heatmap. **(F-K)** Bar plots to visualize the overlap of up- and down-regulated differentially expressed genes (DEGs) between animals treated with **(F)(G)** *arx-2* and LatA, **(H)(I)** *unc-60* and LatA, and **(J)(K)** *lev-11* and LatA, using ggbarstats function in R. Statistical significance of the overlap was assessed using Fisher’s exact test.
